# Rate Aid for Unmarried Mothers

**Published:** 1920-03-27

**Authors:** 


### Rate Aid for Unmarried Mothers.

Hove Corporation has had an application from the Way Servants' Hostel Committee asking the Maternity and Child-welfare Committee to consider the question of sending unmarried mothers and their infants to the Hostel at 21 Seafield Road, paying for them at the rate of 25s. a week till the mother goes to work, and 20s. per week afterwards. The scheme of the Hostel is to admit such mothers and their first infant for at least one year after the child's birth, teaching the mother housewifery and mothercraft, and training her to look after the child. The main object is to keep the mother and child together at least during infancy, and to equip the mother by such training as shall fit her to take her place again as a useful citizen. The mother would go out to daily service for some hours each day when the child could be left. The Hostel is under the charge of a trained-nurse matron. The Maternity Committee is not prepared to recommend the support of a number of cases in the Hostel, but will consider applications in respect of each case as it occurs.

